# Metabolic Dysfunction-Associated Steatotic Liver Disease in the Korean General Population: Epidemiology, Risk Factors, and Non-Invasive Screening

**DOI:** 10.3390/metabo15050299

**Published:** 2025-04-30

**Authors:** Yong Jun Choi, Jooheon Park, Han-Ik Cho, Myung Geun Shin, Eun-Hee Nah

**Affiliations:** 1 Department of Laboratory Medicine, Chonnam National University Hwasun Hospital, Hwasun 58128, Republic of Korea; azarsis@hanmail.net (Y.J.C.);; 2 MEDIcheck LAB, Korea Association of Health Promotion, Seoul 07572, Republic of Korea; hanik@snu.ac.kr; 3 Department of Laboratory Medicine, Seoul National University College of Medicine, Seoul 03080, Republic of Korea; 4 Department of Laboratory Medicine, Chonnam National University Hospital, Gwangju 61469, Republic of Korea

**Keywords:** metabolic dysfunction-associated steatotic liver disease, liver fibrosis, Korean general population, non-invasive testing

## Abstract

**Background/Objectives**: Metabolic dysfunction-associated steatotic liver disease (MASLD) represents a contemporary classification of liver disease linked to metabolic dysfunction. It is recognized as the main form of chronic liver disease and significantly contributes to liver-related morbidity and mortality rates. The epidemiology of MASLD is affected by ethnic background, sex, age, and environmental factors. South Korea is one of the countries that has experienced rapid urbanization. Geographical differences also play a crucial role in the prevalence and progression of the disease. Consequently, it is essential to investigate the prevalence of MASLD; its associated risk factors, particularly in relation to liver fibrosis; and the effectiveness of non-invasive screening techniques within the Korean population. **Methods**: This review describes the prevalence of MASLD, the risk factors related to MASLD with liver fibrosis, and the non-invasive screening approaches suitable for the Korean general population. **Results**: This review underscores the rising incidence and implications of MASLD in South Korea. Notably, among younger demographics, there is a swift increase in both the prevalence of MASLD and its associated risk factors, indicating that MASLD is poised to become a significant public health concern. Non-invasive testing methods are increasingly utilized within at-risk groups to determine the presence of advanced fibrosis. **Conclusions**: Addressing these complex liver diseases necessitates not only ongoing monitoring of MASLD epidemiological patterns but also a unified approach to care that integrates medical interventions with lifestyle changes.

## 1. Introduction

Metabolic dysfunction-associated steatotic liver disease (MASLD) is the updated nomenclature that supersedes the former designation of nonalcoholic fatty liver disease (NAFLD). Since 2023, there has been a notable focus on cardiometabolic dysfunction as a contributing factor to its pathogenesis. Additionally, a more neutral term, “steatotic liver disease” has been adopted to replace the potentially stigmatizing labels of “fatty” or “non-alcoholic”. MASLD is characterized as a hepatic condition marked by the accumulation of fat, which is associated with at least one of five cardiometabolic risk factors: (1) a body mass index (BMI) of 25 kg/m^2^ or higher (23 kg/m^2^ for individuals of Asian descent) or a waist circumference exceeding 94 cm for males and 80 cm for females; (2) fasting blood sugar level of 100 mg/dL (5.6 mmol/L) or higher, postprandial blood sugar levels of 140 mg/dL (7.8 mmol/L) or higher, hemoglobin A1c level of 5.7% (39 mmol/L) or higher, or documented evidence of treatment for type 2 diabetes mellitus (T2DM); (3) blood pressures of 130/85 mmHg or higher, or the use of antihypertensive medications; (4) plasma triglyceride levels exceeding 150 mg/dL (1.70 mmol/L) or the use of medications for dyslipidemia; or (5) plasma high-density lipoprotein cholesterol level of 40 mg/dL (1.0 mmol/L) or lower or the use of treatments for dyslipidemia [1].

The newly established term MASLD encompasses over 96% of existing NAFLD cases [2,3]. Studies suggest that the probability of overlooking liver fibrosis in patients with fatty liver is lower when applying this updated terminology than when using the former NAFLD classification [3]. While there are minor distinctions between the definitions of NAFLD and MASLD, the revised terminology has been employed in the present review.

MASLD includes both uncomplicated hepatic steatosis and metabolic dysfunction-associated steatohepatitis (MASH), which may progress to cirrhosis accompanied by varying stages of liver fibrosis. This condition is recognized as a multi-organ disease that not only leads to liver-related issues such as cirrhosis, end-stage liver disease, and hepatocellular carcinoma (HCC) but also presents a range of extrahepatic complications, including cardiovascular disease (CVD), chronic kidney disease (CKD), and cancers of organs outside the liver [4,5]. The increasing prevalence of obesity, T2DM, and metabolic syndrome worldwide has contributed to the rapid escalation of MASLD, establishing it as the main cause of chronic liver disease [6]. MASLD is frequently underdiagnosed, largely due to the absence of overt symptoms. Nevertheless, the significant impact of liver fibrosis has increased the need for individuals affected by this condition to be detected early. It is also crucial to identify patients who are most susceptible to adverse health consequences.

The epidemiology of MASLD is affected by ethnic background, sex, age, and environmental factors [7]. South Korea is one of the countries that has experienced rapid urbanization. Geographical differences also play a crucial role in the prevalence and progression of the disease. Therefore, it is imperative to understand the prevalence, the associated risk factors of MASLD along with liver fibrosis, and the efficacy of non-invasive screening methods in the Korean general population.

## 2. Epidemiology

MASLD is an increasingly prevalent condition worldwide. This section outlines the epidemiological landscape of MASLD with a focus on global and national trends, associated risk factors, demographic variations, diagnostic variability, the prevalence of lean MASLD, and the natural history of disease progression.

### 2.1. Global and National Prevalence Trends

The global prevalence of MASLD among adults is estimated at approximately 30% [8]. Over the past three decades, a substantial increase has been observed, with prevalence rising from 17.6% in 1990 to 23.4% in 2019, corresponding to an average annual growth rate of approximately 1.0% [8].

In South Korea, the prevalence of MASLD has been reported to range from 10% to 50%, depending on diagnostic modalities and the population cohorts studied (Table 1) [9,10,11,12,13,14,15,16,17,18,19,20,21,22]. According to the Korean National Health Insurance Service (NHIS), the incidence of NAFLD increased from 1.87% in 2010 to 4.47% in 2022, while its prevalence rose from 10.49% to 17.13% during the same period [9]. Based on data from the Korea National Health and Nutrition Examination Survey (KNHANES) I–VII, Kang et al. reported an average annual percentage increase of 2.3%, with projected prevalence expected to reach 39.1% and 43.8% by 2030 and 2035, respectively [10].

Key takeaway: MASLD prevalence is rising significantly both globally and in South Korea, indicating a sustained and accelerating public health concern.

### 2.2. Risk Factors and Demographics

The observed increase in MASLD prevalence parallels the rise in known metabolic risk factors, including general and abdominal obesity, poor dietary habits, and physical inactivity—particularly in individuals under the age of 50 [10]. MASLD is tightly linked to insulin resistance, which contributes to its pathogenesis within the broader spectrum of metabolic syndrome. In South Korea, the age-adjusted prevalence of metabolic syndrome rose from 27.1% in 2001 to 33.2% in 2020, with glycemic dysregulation and abdominal obesity identified as major contributors, especially among men [23].

Key takeaway: Lifestyle-related metabolic disturbances are central to the pathogenesis of MASLD, with a notable impact on younger populations.

### 2.3. Demographic Trends and Regional Differences in MASLD Prevalence

Sex and age significantly influence MASLD epidemiology. Recent Korean data reveal notable demographic disparities in MASLD prevalence, particularly across age and gender. According to a nationwide study using the Health Insurance Review and Assessment Service database, MASLD incidence shows a male predominance in individuals under 50 years, whereas females over the age of 50 exhibited a higher incidence and prevalence of MASLD and MASH [24]. This shift may be related to postmenopausal changes in body composition and hormonal milieu. In younger adults (aged 20–39), there has been a sharp increase in MASLD prevalence, likely linked to rising obesity rates and sedentary lifestyles. Notably, individuals aged 30–49 years showed the highest annual percentage increase in MASLD incidence between 2010 and 2022, as indicated by NHIS data [9]. These trends suggest the need for age-targeted lifestyle interventions and earlier screening programs, particularly for younger males and postmenopausal females, who may represent emerging high-risk groups.

Regional disparities are also emerging. While NAFLD prevalence was similar across urban and rural settings in 2012, urban areas exhibited marginally higher rates by 2022 [9]. Contributing factors include dietary composition, access to healthcare, and physical activity levels. While urban areas generally have better access to healthcare services, they may also present greater exposure to lifestyle risk factors (e.g., poor diet and sedentary behavior), which may outweigh the benefits of healthcare access when it comes to MASLD/NAFLD prevalence. Urban populations tend to consume more high-fat, high-sugar diets and lead more sedentary lifestyles, although sedentary behavior is also increasing in rural populations.

Key takeaway: Epidemiological patterns of MASLD differ by sex, age, and environment, underscoring the need for targeted public health strategies.

### 2.4. Diagnostic Methods and Prevalence Variability

The reported prevalence of MASLD in South Korea varies significantly depending on the diagnostic modalities used, highlighting the influence of methodology on epidemiological data interpretation. Commonly utilized diagnostic tools include abdominal ultrasonography (USG), magnetic resonance imaging proton density fat fraction (MRI-PDFF), liver biopsy, transient elastography (TE), and non-invasive scoring systems such as the fatty liver index (FLI).

USG, the most widely used technique in population-based screenings, is non-invasive, cost-effective, and accessible. However, it lacks sensitivity in detecting mild steatosis (<30% fat content) and is subject to operator variability. In Korean health checkup cohorts, USG-based studies have reported MASLD prevalence rates ranging from 18.7% to 47.9% [11,12,13,14].

MRI-PDFF is considered a gold standard among imaging modalities due to its high sensitivity and ability to quantify hepatic fat content across the entire liver. In a Korean cohort, MRI-PDFF revealed a MASLD prevalence of 29.5% [15], indicating its superior detection accuracy compared to USG. However, its high cost and limited accessibility restrict its routine use in large-scale screenings.

Liver biopsy, though considered the diagnostic gold standard for MASLD and its progressive form, MASH, provides detailed histological information including inflammation and fibrosis. In a study of Korean liver donors, biopsy-based analysis indicated a 51.4% prevalence [16]. However, its invasive nature, sampling error, and ethical limitations make it unsuitable for general population surveillance.

TE, a non-invasive technique that measures liver stiffness and steatosis using vibration-controlled transient ultrasound, offers a reliable and rapid assessment of hepatic fat and fibrosis. A Korean study using this method reported a 42.9% prevalence [17]. While TE is increasingly used in clinical practice, limited availability and operator dependency may affect consistency.

Lastly, FLI, which integrates anthropometric and biochemical parameters, provides a non-invasive, easily calculable screening tool. Despite its convenience, it has lower diagnostic accuracy for steatosis, as reflected by a much lower MASLD prevalence of 12.6% in a Korean cohort [18].

These wide variations in prevalence estimates underscore the challenges of standardizing MASLD epidemiological data. Differences in diagnostic sensitivity, specificity, and application context (e.g., clinical vs. screening settings) can lead to an over- or underestimation of disease burden. Therefore, comparisons across studies must carefully consider the diagnostic tools employed. The harmonization of diagnostic criteria and validation of non-invasive modalities against biopsy or MRI-PDFF in Korean populations are essential for improving accuracy and comparability in future MASLD research.

Key takeaway: Prevalence estimates of MASLD are highly dependent on the diagnostic tools used; consistent criteria are necessary for meaningful comparisons.

### 2.5. Lean MASLD

Although MASLD is frequently associated with obesity, its presence in non-obese individuals—referred to as lean NAFLD—is increasingly recognized. Global prevalence estimates for lean NAFLD range from 3.8% to 34.1% [25]. In Korean cohorts with BMI below 23–25 kg/m^2^, prevalence rates range from 3.7% to 17.6% [19,20,21,22].

Key takeaway: MASLD can affect non-obese individuals, highlighting the importance of evaluating metabolic health regardless of BMI.

### 2.6. Lifestyle, Disease Progression, and Long-Term Outcomes

Unhealthy lifestyle choices—particularly excessive caloric intake and low physical activity—are key contributors to hepatic fat accumulation and MASLD development. This can lead to hepatocellular lipotoxicity, inflammation, oxidative stress, and fibrogenesis. Hepatic steatosis may progress to MASH, cirrhosis, and eventually HCC. Disease progression is often asymptomatic and can span several years. Studies have shown that 10–25% of individuals with steatosis progress to MASH, 5–8% of MASH patients develop cirrhosis within five years, and 12.8% of patients with cirrhosis develop HCC within three years [26,27,28,29,30,31,32]. From 2010 to 2022 in Korea, the incidence of NAFLD-related cirrhosis and HCC was 2.22% and 0.77%, respectively [9]. Another study reported an annual cirrhosis incidence rate of up to 2.6% [33]. A nationwide cohort study indicated that the risk of HCC was 2.2-fold, 2.3-fold, and 4.7-fold higher in individuals with resolved, incident, and persistent MASLD, respectively, compared to those without MASLD [34].

Key takeaway: MASLD can progress to severe liver complications, including cirrhosis and HCC. Early identification and fibrosis staging are critical in disease management.

The prevalence of MASLD is increasing globally and in South Korea, driven by a rise in metabolic risk factors, lifestyle changes, and demographic shifts. Diagnostic heterogeneity contributes to variable prevalence estimates, and non-obese individuals are not exempt from risk. MASLD can progress silently to advanced liver disease, including cirrhosis and hepatocellular carcinoma. These findings underscore the urgency for standardized diagnostic practices, early screening, and comprehensive interventions targeting modifiable risk factors to mitigate the growing public health burden of MASLD.

## 3. Pathogenesis

MASLD is a complex and dynamic process that involves multifaceted interactions between metabolic, genetic, and environmental factors. Traditionally considered a consequence of insulin resistance and obesity, recent studies have proposed novel mechanisms that offer a broader understanding of how hepatic lipid accumulation initiates and drives liver injury.

### 3.1. Hepatic Lipid Accumulation: Causes and Consequences

Hepatic steatosis, the hallmark of MASLD, results from an imbalance between lipid acquisition and disposal in hepatocytes. Excessive lipid accumulation occurs due to increased free fatty acid (FFA) influx from adipose tissue, enhanced de novo lipogenesis, impaired β-oxidation, and reduced lipid export via very low-density lipoproteins [29]. Insulin resistance plays a central role by upregulating DNL through sterol regulatory element-binding protein-1c (SREBP-1c) while simultaneously failing to suppress lipolysis in peripheral adipose tissues, thereby increasing FFA delivery to the liver [30]. This accumulation of triglycerides, although initially protective, can eventually trigger lipotoxicity—a state in which non-triglyceride lipid species such as ceramides, diacylglycerols, and free cholesterol induce hepatocyte dysfunction, endoplasmic reticulum (ER) stress, mitochondrial injury, and oxidative damage, ultimately progressing to inflammation, fibrosis, and hepatocellular death [31].

### 3.2. Regulatory Factors of Hepatic Lipid Metabolism

Several molecular regulators control hepatic lipid accumulation. These include transcription factors (SREBP-1c, ChREBP), nuclear receptors (PPARα/γ, FXR), and signaling pathways such as AMPK and mTOR. The dysregulation of these factors in MASLD leads to aberrant lipid synthesis and defective lipid catabolism. In addition, adipokines (e.g., adiponectin, leptin) and pro-inflammatory cytokines (e.g., TNF-α, IL-6) secreted from dysfunctional adipose tissue further exacerbate hepatic lipid storage and promote insulin resistance [32].

### 3.3. Lipotoxicity and Its Mechanisms

Lipotoxicity is a pivotal link between simple steatosis and progressive liver damage in MASLD. Toxic lipid metabolites disrupt mitochondrial function, enhance reactive oxygen species production, and initiate apoptotic and necroptotic pathways. Moreover, ER stress induced by lipid overload activates the unfolded protein response, which can switch from adaptive to pro-apoptotic signaling under persistent stress. Kupffer cell activation and hepatic stellate cell stimulation in response to hepatocyte injury further perpetuate inflammation and fibrogenesis [33].

### 3.4. Gut Microbiota and Hepatic Lipid Metabolism

Recent insights have highlighted the gut–liver axis as a critical player in MASLD pathogenesis. Dysbiosis, or alteration in gut microbiota composition, leads to increased intestinal permeability and translocation of bacterial endotoxins (e.g., lipopolysaccharide), which activate hepatic Toll-like receptors and promote inflammatory cascades. Moreover, certain gut microbes influence hepatic lipid metabolism directly by modulating bile acid profiles and producing short-chain fatty acids that affect lipogenesis and insulin sensitivity. For instance, reduced *Akkermansia muciniphila* and elevated Firmicutes/Bacteroidetes ratios have been linked to worsened hepatic steatosis [34].

### 3.5. Ferroptosis and Lipid Peroxidation in MASLD

Ferroptosis is an iron-dependent form of regulated cell death characterized by the accumulation of lipid peroxides. In MASLD, excess intracellular iron catalyzes lipid peroxidation via Fenton reactions, particularly when antioxidant defenses such as glutathione peroxidase 4 are overwhelmed. The resulting oxidative stress damages hepatocyte membranes and organelles, contributing to cell death and inflammation. Notably, ferroptosis serves as a mechanistic bridge linking lipid overload, oxidative injury, and fibrogenesis in MASLD [35].

### 3.6. Genetic Predisposition in the Korean Population

Genetic susceptibility plays a critical role in the development and progression of MASLD, with numerous studies highlighting population-specific variants. In the Korean population, several genome-wide association studies (GWASs) and candidate gene studies have identified genetic loci associated with increased hepatic fat accumulation and MASLD risk, mirroring—but also diverging from—patterns seen in Western cohorts.

The patatin-like phospholipase domain-containing 3 (*PNPLA3*) rs738409 (I148M) is one of the most consistently replicated genetic risk factors for MASLD globally. In Koreans, the G allele (coding for methionine) is significantly associated with increased hepatic fat content, elevated alanine aminotransferase (ALT) levels, and greater risk for progression to steatohepatitis and fibrosis. The minor allele frequency of rs738409 is notably higher in East Asians, including Koreans, compared to Europeans, suggesting a greater genetic predisposition to MASLD in this ethnic group [36].

While globally validated variants such as *PNPLA3* rs738409 contribute significantly to MASLD susceptibility in Koreans, other variants like *GCKR* rs780094 and novel loci identified through the Korean Genome and Epidemiology Study (KoGES) offer unique insight into population-specific genetic risks. These findings reinforce the need for personalized approaches to MASLD risk assessment and management in Korean populations, incorporating both genetic background and lifestyle factors.

In summary, MASLD pathogenesis extends beyond simple lipid accumulation to encompass a spectrum of lipotoxic insults, inflammatory cascades, and cell death pathways such as ferroptosis. Emerging insights into the roles of gut microbiota and lipid peroxidation mechanisms offer promising avenues for targeted therapies and early intervention. Understanding these novel perspectives is essential to advancing MASLD research and developing precision-based treatments.

## 4. Underlying Causes and Contributing Factors of MASLD in the Korean Population

The burden of MASLD is rapidly rising in Korea, in parallel with increasing rates of obesity, metabolic syndrome, and T2DM. While the core pathophysiological mechanisms of MASLD are shared globally, several factors unique to the Korean population influence disease prevalence, progression, and clinical characteristics.

### 4.1. Epidemiologic Shifts and Lifestyle Changes

In recent decades, Korea has experienced rapid urbanization and Westernization of dietary patterns. Traditional Korean diets, once rich in vegetables and low in fat, have shifted toward higher consumption of processed foods, red meats, and sugar-sweetened beverages. This dietary transition, coupled with increasingly sedentary lifestyles, has contributed to a rising prevalence of obesity and metabolic disorders—key drivers of MASLD. Data from the KNHANES indicate a steady rise in MASLD prevalence, particularly among younger adults. The condition, once predominantly seen in middle-aged individuals, is now being detected in adolescents and young adults due to early-onset obesity and insulin resistance [10].

### 4.2. Genetic and Ethnic Susceptibility

Genetic predisposition plays a significant role in MASLD susceptibility. Among East Asians, including Koreans, the *PNPLA3* I148M variant has been consistently associated with increased hepatic fat accumulation and fibrosis. Moreover, emerging evidence suggests that the *TM6SF2* E167K and *MBOAT7* polymorphisms, though less frequent, may also modulate disease severity [36]. Interestingly, Koreans with MASLD often present with lower BMI compared to Western populations, indicating a “lean MASLD” phenotype. This suggests that Korean individuals may be more susceptible to hepatic steatosis at relatively lower levels of adiposity, possibly due to differences in visceral fat distribution or insulin sensitivity.

### 4.3. Metabolic Syndrome and Insulin Resistance

Metabolic syndrome, encompassing central obesity, hypertension, dyslipidemia, and insulin resistance, is a well-recognized driver of MASLD. In Korea, metabolic syndrome affects nearly one-third of adults, with increasing prevalence in both sexes. Notably, Korean men tend to exhibit higher rates of visceral adiposity and insulin resistance despite having a normal BMI, predisposing them to more aggressive forms of MASLD. The increasing incidence of T2DM in Korea—now affecting over 14% of adults—further fuels MASLD progression and fibrosis risk. Importantly, many Korean patients with T2DM remain undiagnosed or suboptimally managed, exacerbating liver-related morbidity [23].

### 4.4. Sociocultural and Healthcare Factors

Stigma around obesity and liver disease, especially among younger individuals, may lead to delayed diagnosis and underreporting. Furthermore, liver function tests and ultrasound screenings are not consistently included in routine health checks, contributing to the underdiagnosis of MASLD in its early stages. Despite universal health coverage, disparities in health literacy and access to specialty care persist, especially in rural regions [9,11].

## 5. Liver Fibrosis in MASLD

The progression of liver fibrosis in MASLD is linked not only to the development of cirrhosis but also to a higher risk of overall mortality. A key therapeutic objective is therefore is to prevent or even reverse the progression of liver fibrosis. The early identification of liver fibrosis followed by subsequent evaluations and the monitoring of treatment efficacy requires dependable non-invasive test (NIT) techniques. Effectively addressing the increasing prevalence of MASLD will reduce the rate of it progressing to cirrhosis or liver cancer, thus reducing mortality rates and also the associated healthcare challenges.

The classification of liver fibrosis into stages F0 to F4 (F0, normal; F1, mild fibrosis; F2, moderate fibrosis; F3, advanced fibrosis; F4, cirrhosis) identifies patients with F2 or higher as the primary candidates for pharmacological intervention. Conversely, individuals with F1-stage liver disease have the potential to revert to a normal state through lifestyle changes and the management of related metabolic conditions. The precise monitoring and diagnosis of liver fibrosis through NIT techniques is of paramount importance.

### 5.1. Prevalence and Risk Factors of Liver Fibrosis

The health screening conducted among the general population in Korea reveals that the prevalence of significant liver stiffness, categorized as stage F2 or higher, is between 9% and 13% [37,38]. There is a positive correlation between the severity of fatty liver and the presence of fibrosis [13,14]. Findings from extensive health screening facilities in Korea show that 8.35% and 2.04% of individuals with fatty liver exhibit fibrosis at stages ≥F2 and ≥F3, respectively [37]. The presence of T2DM is associated with an increase in both the prevalence and the severity of fibrosis, particularly at stage F3 or higher. Among the various criteria used to identify at-risk individuals in the Korean population, those with diabetes demonstrated the highest rate of advanced hepatic fibrosis at 8.8%. In comparison, elevated aminotransferase levels (6.0%), the presence of two or more metabolic risk factors (3.1%), and hepatic steatosis identified via sonography (2.3%) were linked to the highest prevalence of hepatic fibrosis [38]. It is noteworthy that 28.2% to 41.1% of individuals within these at-risk groups did not exhibit fatty liver on ultrasound [38,39]. Consequently, it is advisable that screening for hepatic fibrosis in primary care settings be expanded to include individuals with a metabolically unhealthy status, beyond those diagnosed with a fatty liver.

### 5.2. Diagnosis of Liver Fibrosis

The stage of liver fibrosis is a critical determinant of liver-related diseases and mortality and serves as a significant indicator for the occurrence of comorbid conditions such as diabetes and CVD [40], which makes it essential to accurately diagnose liver fibrosis.

#### 5.2.1. Liver Biopsy

Liver biopsy is the gold standard for diagnosing MASLD and conducting histological evaluations. It remains necessary for assessing fibrosis in high-risk patients with advanced liver fibrosis during long-term follow-up and for the development and efficacy evaluation of MASH therapeutics. Various scoring systems are used to classify the histological stages of liver disease. In the context of MASLD, the Nonalcoholic Steatohepatitis Clinical Research Network (NASH CRN) scoring system is commonly employed, which differs from the Metavir score designed for classifying fibrosis due to viral chronic hepatitis [41,42]. The NASH CRN scoring system includes the following stages: F1, mild fibrosis localized to the perivenular and periportal areas; F2, moderate fibrosis that extends to the portal vein and adjacent regions; F3, severe fibrosis characterized by multiple bridging fibrous septa connecting portal areas; and F4, cirrhosis, where numerous fibrous septa create partitions within liver tissue. In clinical settings, significant fibrosis is classified as stage ≥F2, while advanced fibrosis is identified as stage ≥F3.

#### 5.2.2. Non-Invasive Diagnostic Methods for Liver Fibrosis

Liver biopsies present several limitations when utilized for the early screening of MASLD and monitoring disease progression. NITs have become increasingly important for evaluating liver fibrosis in individuals suspected of having MASLD by providing a safer and less invasive alternative to traditional liver biopsy. These NITs may also be useful in early detection and management strategies, thereby decreasing the reliance on biopsy procedures. NITs can be broadly categorized into biological approaches that quantify serum biomarkers and physical approaches that measure liver stiffness [43]. By integrating these two methodologies, it is anticipated that NITs could effectively substitute liver biopsies in the diagnosis of liver fibrosis. An overview of the cut-off of the different NITs related to MASLD-induced liver fibrosis is presented in Table 2.

##### Vibration Controlled Transient Elastography (VCTE)

VCTE is the most commonly employed diagnostic technique for evaluating liver tissue. This method measures the stiffness of the liver, which is correlated with the extent of liver fibrosis. VCTE is also effective in ruling out significant liver fibrosis and in monitoring the progression of liver disease. Although there is no consensus in clinical practice for the liver stiffness measurement (LSM) cut-off values for excluding advanced fibrosis, a threshold of 8 kPa is the most widely validated and has yielded a negative predictive value (NPV) exceeding 90% [44]. Values exceeding 12 kPa are associated with a significant increase in the probability of advanced fibrosis, necessitating referral for these patients [45]. Additionally, a meta-analysis indicated that LSM values higher than 12–15 kPa may serve as indicators of advanced fibrosis [46].

##### Magnetic Resonance Elastography (MRE)

MRE is a quantitative imaging modality that utilizes magnetic resonance technology to evaluate the stiffness of hepatic tissue. The diagnostic thresholds for advanced hepatic fibrosis identified through MRE exhibit considerable variability across different studies. The most commonly referenced threshold for diagnosing advanced fibrosis is 3.6 kPa [47,48]. This technique provides numerous benefits, such as reduced examination duration and the capability to assess stiffness in the early phases of NAFLD, along with superior diagnostic accuracy when compared to VCTE. Nonetheless, the high expense associated with MRE poses a significant barrier to its implementation as a standard screening method for NAFLD, making it more appropriate for use in clinical research settings.

##### Non-Invasive Score Calculations to Detect Liver Fibrosis

A widely utilized approach for predicting liver fibrosis is analyzing the results of clinical and laboratory test. Several serum markers and scoring systems have been proposed to evaluate fibrosis severity, including the NAFLD fibrosis score (NFS), Fibrosis-4 Index (FIB-4), AST-to-platelet-ratio index (APRI), AST-to-ALT ratio (AAR), and enhanced liver fibrosis (ELF) test. The APRI score is a simple tool for predicting liver fibrosis [49]. NFS includes variables such as age, BMI, AAR, platelet count, hyperglycemia, and albumin level, while FIB-4 combines age, AST, ALT, and platelet count. Two sets of cut-off values have been established: one with high sensitivity (1.3 for FIB-4 and −1.455 for NFS) and another with high specificity (3.25 for FIB-4 and 0.676 for NFS) [50]. In primary care, FIB-4 is categorized into three ranges: <1.30, 1.30–2.67, and >2.67. FIB-4 < 1.30 has an NPV of 90% for excluding advanced fibrosis, making it suitable for low-risk management in primary care, focusing on obesity and CVD prevention [51]. Age is an important factor, prompting higher cut-offs for those over 65 years (2.0 for FIB-4 and 0.12 for NFS) [52]. For Korean general population under 45 years, APRI has higher screening efficacy than FIB-4 and NFS [53].

Thresholds for diagnosing fibrosis can vary based on population risk factors. A study in South Korea used an FIB-4 cut-off of 1.3 to identify advanced liver fibrosis, yielding a sensitivity of 66.7% (confidence interval of 50–78%), NPV of 99.0% (confidence interval of 96–99%), and an accuracy of 77.9% (confidence interval of 76–77%). Lowering the FIB-4 cut-off to 1.0 improved sensitivity to 82.6%, while NPV remained at 99.2%. Thus, FIB-4 1.0 is recommended for general screening [54]. FIB-4 is more effective than NFS in identifying advanced fibrosis [55] and is useful for at-risk groups (e.g., those with diabetes, liver abnormalities, or metabolic risk factors) in the Korean population [37,56]. The ELF blood test evaluates three specific extracellular matrix proteins: hyaluronic acid, amino-terminal propeptides of type III procollagen, and tissue inhibitors of metalloproteinase-1 [57]. An ELF score of <7.7 indicates a low risk of advanced fibrosis, allowing effective management in primary care settings. Conversely, patients with intermediate scores from 7.7 to 9.8 as well as those with high-risk scores exceeding 9.8 should be referred to hepatology specialists for further evaluations and potentially also liver biopsies [58,59].

The recently developed Mac-2-binding protein glycosylated isomer (M2BPGi) test represents a specific diagnostic tool for identifying liver fibrosis. Mac-2-binding protein (M2BP) acts as a messenger from Kupffer cells and is secreted by hepatic stellate cells. Fibrosis induces glycosylation the N-glycan attached to M2BP, resulting in the formation of M2BPGi. This compound can be detected based on specific reactions with lectins, making it possible to use a blood test to assess both the presence and severity of liver fibrosis. Using a cutoff of 0.75 for stage ≥F3 in the M2BPGi test yielded a sensitivity of 80.0%, a specificity of 77.9%, and an NPV of 98.9%, thereby providing a significant advantage in the early detection of fibrosis progression in liver diseases in Korean health screening setting [60]. Furthermore, M2BPGi has been correlated with changes in liver fat as measured by MRI-PDFF in individuals with NAFLD undergoing lifestyle interventions in a Korean population [61].

Establishing a single threshold that achieves a perfect balance between sensitivity and specificity is challenging, and there is a lack of clear criteria for interpreting results. Consequently, assessments of fibrosis are often supported by extensive clinical experience and serve as an adjunct to imaging findings.

**Table 2:** Overview of the cutoff of the different non-invasive tests (NITs) for advanced liver fibrosis in metabolic dysfunction-associated steatotic liver disease.

NIT	Risk /Fibrosis Stage	Cut-Off	Rule Out Advanced Fibrosis *	References
FIB-4	Low	<1.3	<1.3	[51]
	Intermediate	1.3–2.67		
	High	>2.67		
ELF	Low	<7.7	<9.8	[58,59]
	Intermediate	7.7–9.8		
	High	>9.8		
LSM	Low	<8 kPa	<8 kPa	[44]
	Intermediate	8–12 kPa		
	High	>12 kPa		
MRE	≥F3	>3.6 kPa	Not established	[47,48]
NFS	≥F3	>−1.455	<−1.455	[50]
APRI	≥F1	>0.45	Not established	[49]
M2BPGi	≥F3	>0.75	Not established	[60]

* The cutoffs are recommended to rule out advanced fibrosis in clinical practice (2021 update on EASL clinical practice guidelines). Abbreviations: FIB-4, fibrosis-4 Index; ELF, enhanced liver fibrosis; LSM, liver stiffness measurement; MRE, magnetic resonance elastography; NFS, nonalcoholic fatty liver disease fibrosis score; APRI, aspartate aminotransferase-to-platelet-ratio index; M2BPGi, Mac-2-binding protein glycosylated isomer.

## 6. Extrahepatic Outcomes of MASLD

The MASLD patients had a higher prevalence of comorbidities, such as hypertension, osteoarthritis, diabetes, and fractures of osteoporosis. Data from the Korean NHIS showed that the most common comorbid condition among NAFLD patients was hypertension, present in 42.3% of the cases, followed by osteoarthritis, diabetes, and fractures or osteoporosis, seen, respectively, in 25.7%, 19.1%, and 15.2% of all NAFLD subjects in 2022 [9]. The primary risk factors for MASLD are metabolic in nature, including obesity, insulin resistance, T2DM, hypertension, and dyslipidemia. The prevalence of hypertension, T2DM, and dyslipidemia was also higher in participants with MASLD as compared to no SLD, seen, respectively, in 40.9%, 14.6%, and 25.0% of MASLD in a Korean nationwide cohort study [62]. Liver-induced low-grade systemic inflammation in MASLD has the potential to induce cardiovascular inflammation, atherosclerosis, CKD, and various forms of cancer affecting multiple organs [63].

### 6.1. Obesity in MASLD

Obesity is recognized by the World Health Organization as a global epidemic due to its high and increasing prevalence. Approximately 80% of individuals with MASLD are obese, and those with obesity face 4.6-, 4.1-, and 1.89-fold higher risks of developing MASLD, liver cirrhosis, and liver cancer, respectively. Obesity and abdominal obesity prevalence have increased for the entire population from 2009 to 2019 in the obesity fact sheet in Korea [64]. Obesity prevalence has risen rapidly in individuals in their 20s and 80s compared with other age groups in Korea. Additionally, the prevalence of class II obesity, defined as a BMI of 35.0 kg/m^2^ or higher, has surged significantly, with an approximate threefold increase observed in both male and female populations.

### 6.2. T2DM in MASLD

T2DM is diagnosed in 22.5% and 43.6% of individuals with MASLD and MASH, respectively. Furthermore, there is a significant risk of developing new-onset T2DM in 80% of MASLD patients, with MASH patients exhibiting a threefold higher risk. T2DM also accelerates the progression of MASLD toward cirrhosis or HCC [65,66]. According to fatty liver and diabetes statistics in Korea (nationwide data from 2009 to 2017), the prevalence of severe fatty liver disease (by fatty liver index ≥ 60) was higher in individuals with T2DM, accounting for 29.6% in 2017. In the young population (20–39 years) with T2DM, more than half (60.1%) had severe fatty liver disease. Additionally, the prevalence of severe fatty liver disease in the young population with T2DM has increased sharply compared with the other age groups with T2DM [67]. Considering the growing burden of fatty liver disease in the young age, particularly people with T2DM, an early screening strategy should be considered in these Korean populations. Similarly, it has been known that individuals with both NAFLD and T2DM showed a significantly higher prevalence of chronic vascular complications of T2DM, including coronary, cerebral, and peripheral vascular disease in Korea [68,69]. In this respect, high-risk patient identification, and early intervention are important to decrease future disease burden in Korea.

### 6.3. Cardiovascular Complications in MASLD

Epidemiological studies indicate that MASLD significantly increases the long-term risk of CVD, particularly in high-risk populations susceptible to conditions such as ischemic heart disease, cardiomyopathy, and arrhythmias. This higher risk ultimately contributes to the increased morbidity and mortality associated with CVD. Furthermore, the risk of CVD is markedly higher in individuals with more advanced stages of MASLD and liver fibrosis [70,71]. A comprehensive analysis utilizing a nationwide health screening database from Korea, with a median follow-up duration of 9.0 years, revealed that the prevalence of MASLD was 47.2%, while the incidence rate of CVD was recorded at 8.5 per 1000 person-years. The presence of MASLD was found to elevate the risk of CVD in comparison to individuals without steatotic liver disease [62]. Furthermore, an additional study conducted in Korea examined the cardiovascular consequences of MASLD, the accumulation of cardiometabolic risk factors, and their changes over time. With a median follow-up period of 12.3 years, the findings indicated a progressive increase in CVD risk correlated with a higher count of cardiometabolic risk factors. Notably, the emergence of MASLD during the follow-up period was linked to an increased risk of CVD, whereas a regression in MASLD was associated with a decreased risk of CVD in a Korean cohort study [72].

### 6.4. Malignant Complications in MASLD

Liver-induced low-grade systemic inflammation is also implicated in facilitating the development of cancer in multiple organs [73]. The association of MASLD with extrahepatic cancers has been documented, particularly malignant tumors of the gastrointestinal tract such as those affecting the colon, esophagus, and stomach. Additionally, there is an increased risk of metastatic cancers originating from primary tumors of unknown etiology, as well as increased susceptibilities to renal cancer in males, breast cancer in females, and lung cancer and melanoma in both sexes [74]. Numerous nationwide cohort studies conducted in Korea have indicated a correlation between MASLD and an elevated risk of extrahepatic malignancies, including colorectal cancer, biliary tract cancers, renal cancer, and thyroid cancer [75,76,77,78,79]. Furthermore, one such study revealed that individuals with the diabetic or lean subtype of MASLD exhibited a greater risk of developing extrahepatic malignancies compared to those without MASLD.

### 6.5. CKD in MASLD

The prevalence rate of CKD is 20–50% higher in individuals with MASLD regardless of the presence of risk factors such as hypertension or T2DM [80]. Furthermore, a meta-analysis found that MASLD increases the prevalence and incidence of CKD by five- and threefold, respectively [81]. A study involving the Korean general population at primary facilities found that the prevalence rate of MASLD was 12.4%. The occurrence of albuminuria was noted at 16.2%, while proteinuria was observed at 8.0%, both of which were more common in individuals with NAFLD. Furthermore, NAFLD demonstrated an independent association with CKD of at least mild severity and was linked to significant liver fibrosis, as assessed through MRE in patients with NAFLD [82].

## 7. Management of MASLD

The management of MASLD primarily emphasizes lifestyle modification, with weight reduction through dietary changes and regular physical activity serving as the cornerstone of intervention. Clinical evidence demonstrates that a 3–5% reduction in body weight can significantly reduce hepatic steatosis, while a ≥10% weight loss may lead to the resolution of steatohepatitis and regression of fibrosis [83]. While these outcomes highlight the effectiveness of lifestyle intervention, long-term adherence to such behavioral changes remains a critical barrier. This challenge has led to increasing interest in structured dietary strategies and personalized lifestyle approaches based on individual risk profiles.

One emerging approach involves the Korean adaptation of the Mediterranean diet. A recent genome-wide interaction study utilizing data from the KoGES investigated how adherence to the Mediterranean diet modulates genetic risk for MASLD [84]. This study identified significant gene–diet interactions, particularly with variants in genes such as *GCKR*, *EIF2B4*, *IFT172*, and *ZMAT3*. Notably, carriers of the rs780094 variant near the *GCKR* gene exhibited a reduced risk of MASLD when adhering closely to the Mediterranean diet. These findings underscore the potential of dietary modulation to mitigate MASLD risk in genetically predisposed individuals, emphasizing the importance of culturally adapted dietary guidelines as part of MASLD management strategies. Despite the promise of lifestyle-based approaches, pharmacological therapy remains an area of active development, especially for patients unable to achieve sufficient weight loss or who present with advanced disease. To date, no medications have received regulatory approval specifically for MASLD or MASH based on the NASH CRN criteria, which require at least a one-stage improvement in fibrosis without the worsening of steatohepatitis.

Among off-label options, vitamin E has shown histologic improvement in non-diabetic patients with biopsy-proven MASH [85]. Additionally, antidiabetic medications—including pioglitazone, GLP-1 receptor agonists (e.g., liraglutide, semaglutide) [86], and SGLT2 inhibitors [87]—have demonstrated efficacy in reducing hepatic fat and improving insulin resistance. Emerging agents such as dual incretin agonists (e.g., tirzepatide) [88] are also being evaluated in MASLD populations with coexisting T2DM or obesity and show promise for future therapeutic use.

## 8. Conclusions and Future Directions

This review highlights the growing clinical and public health burden of MASLD in South Korea, where prevalence rates have steadily increased over recent decades, particularly among younger adults and non-obese individuals. This analysis of epidemiological data reveals that the projected trajectory of MASLD prevalence—potentially exceeding 40% by 2035—parallels rising rates of obesity, abdominal obesity, sedentary behavior, and poor dietary habits within the Korean population. A notable finding is the heterogeneity in MASLD prevalence based on diagnostic modality, region (urban vs. rural), sex, and age, reflecting the influence of both lifestyle and genetic factors in disease susceptibility. Furthermore, genetic variants such as *PNPLA3* and *GCKR* and gene–diet interactions (e.g., with the Mediterranean diet) have emerged as critical modifiers of MASLD risk, underscoring the need for personalized prevention strategies in Korea. Clinically, the early identification of individuals at risk for advanced liver fibrosis remains the cornerstone of MASLD management. The increasing adoption of non-invasive fibrosis assessment tools offers a practical means to stratify risk and initiate timely interventions in both primary and specialized care settings. Nevertheless, sustained lifestyle modification—including weight loss, improved dietary quality, and increased physical activity—remains the most effective intervention for MASLD management.

Looking forward, future directions should focus on population-level surveillance to monitor MASLD trends across age, region, and socioeconomic groups; the integration of genetic risk profiling into clinical practice to guide personalized interventions; the development and validation of culturally adapted lifestyle interventions, including dietary strategies; and the expansion of pharmacologic options through ongoing clinical trials targeting fibrosis resolution and metabolic correction.

In conclusion, MASLD represents an emerging metabolic liver disease of major importance in Korea. A coordinated approach that combines risk stratification, non-invasive diagnostics, metabolic comorbidity management, and tailored lifestyle interventions will be essential in curbing the burden of MASLD across the Korean population.

## Figures and Tables

**Table 1:** Prevalence of NAFLD/MASLD in the Korean general population according to study cohorts, diagnostic modalities, and demographic parameters.

Study Cohorts	Diagnostic Method	Prevalence				References
		General	Gender	Environment	BMI	
Data from KNHANES (1998–2017)	HSI	18.9–24.2%				[10]
Health checkup visitors (2000)	USG	31.60%			14.40% (<25 kg/m^2^)	[19]
Ansung-Ansan cohort (2001–2002)	FLI	12.62%				[18]
Health checkup visitors (2003)	USG	18.70%	23.0% (M) /13.7% (F)			[11]
Potential liver donors (2004–2005)	Biopsy	51.40%				[16]
Health checkup visitors (2008–2010)	USG	27.10%			17.60% (<23 kg/m^2^)	[21]
Health checkup visitors (2009–2010)	USG	27.30%	38.3% (M) /12.6% (F)			[12]
Data from NHIS (2010–2022)	USG	10.49–17.13%		17.15% (urban)/15.95% (rural)		[9]
Health checkup visitors (2013–2014)	TE	42.90%				[17]
Health checkup visitors (2014)	USG	27.20%			12.40% (<25 kg/m^2^)	[20]
Health checkup visitors (2017–2020)	USG	33.90%				[13]
Health checkup visitors (2017–2020)	MRI-PDFF	29.51%				[15]
Health checkup visitors (2018–2020)	USG	47.90%				[14]
Health checkup visitors (2018–2020)	USG	35.20%			3.70% (<23 kg/m^2^)	[22]

Abbreviations: NAFLD, nonalcoholic fatty liver disease; MASLD, metabolic dysfunction-associated steatotic liver disease; BMI, body mass index; KNHANES, Korea National Health and Nutrition Examination Survey; NHIS, National Health Insurance Service; HSI, hepatic steatosis index; USG, ultrasonography; FLI, fatty liver index; TE, transient elastography; MRI-PDFF, magnetic resonance imaging proton density fat fraction.

## Data Availability

No new data were created or analyzed in this study.
